# Supplementary material for: HIF-1α and Pro-Inflammatory Signaling Improves the Immunomodulatory Activity of MSC-Derived Extracellular Vesicles
Source: Int J Mol Sci. 2021 Mar 26;22(7):3416. doi: 10.3390/ijms22073416 (PMC8036951; doi:10.3390/ijms22073416)
Supplement: Supplementary file 1 [file ijms-22-03416-s001.pdf]

Gómez-Ferrer, *et. al.* Supplementary figure 1

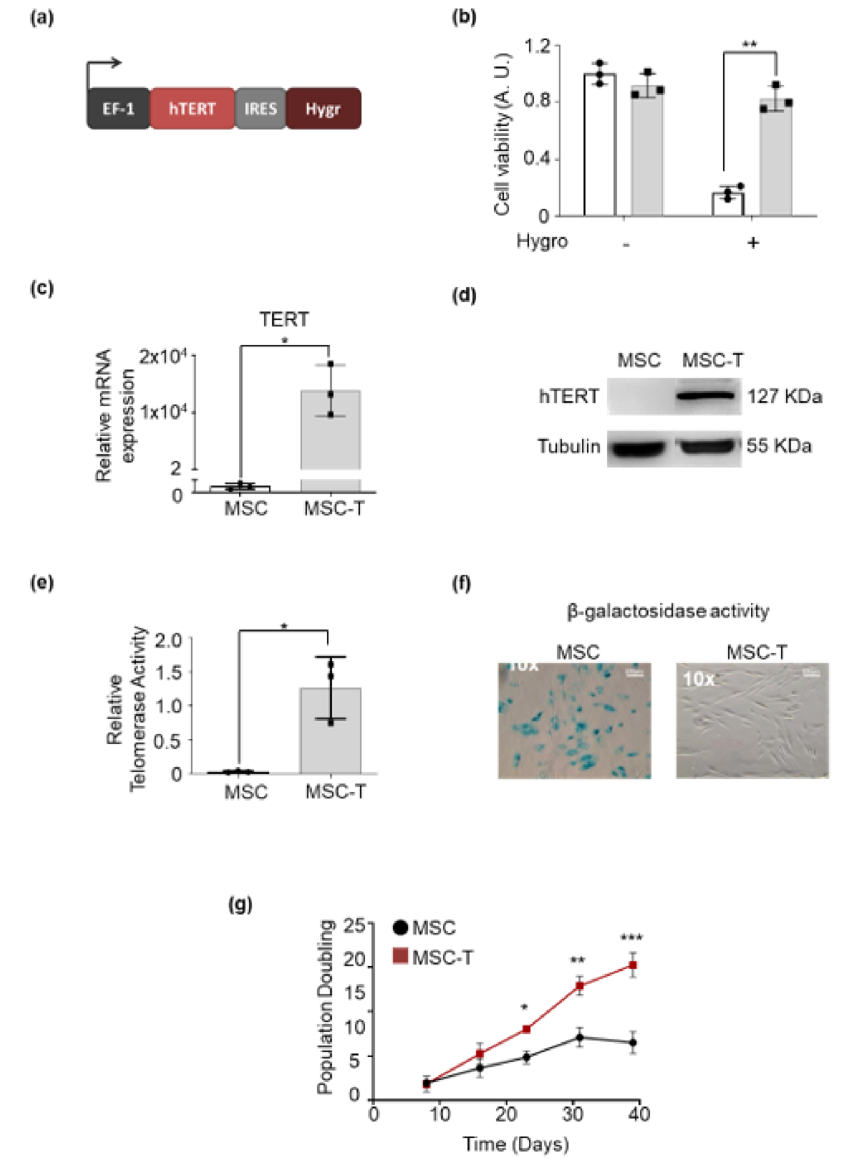

Scheme 1. Generation and validation of a stable and non-senescent cell line. a) Schematic representation of pLV-hTERT-Hygromyn lentiviral vector. b) Quantification of viable MSC (black) and MSC-T (grey) detected by MTT assay treated or not with hygromycin for 48 h. Values are represented as relative to MSC not treated condition and graphs represent the mean ± SD of 3 independent experiments. Paired t-test was used for statistics. c) hTERT gene expression levels quantified by RT-qPCR in MSC (black) and MSC-T (grey). The expression levels of the target gene in each sample were normalized to GAPDH expression. Values are represented as relative to MSCs levels. Graphs represent mean ± SD of 3 independent experiments. Paired t-test was used for statistics. d) Representative western blot of hTERT protein; α-tubulin was used as a loading control. e) Relative telomerase activity (RTA) by relative-quantitative telomerase repeat amplification protocol assay (RQ-TRAP) in 1x106 MSC (black) and MSC-T (grey). Graphs represent the mean ± SD of 3 independent experiments. Paired t-test was used for statistics. f) Representative images of senescence associated β-galactosidase detection in MSC (left) and MSC-T (right) at passage 14. g) Proliferation assay. MSC (black) and MSC-T (red) growth curve; represented by doubling population at different timepoints. Each point represents the mean ± SD of 3 independent experiments. Paired t-test was used for statistics. *p < 0.05, ***p < 0.001.

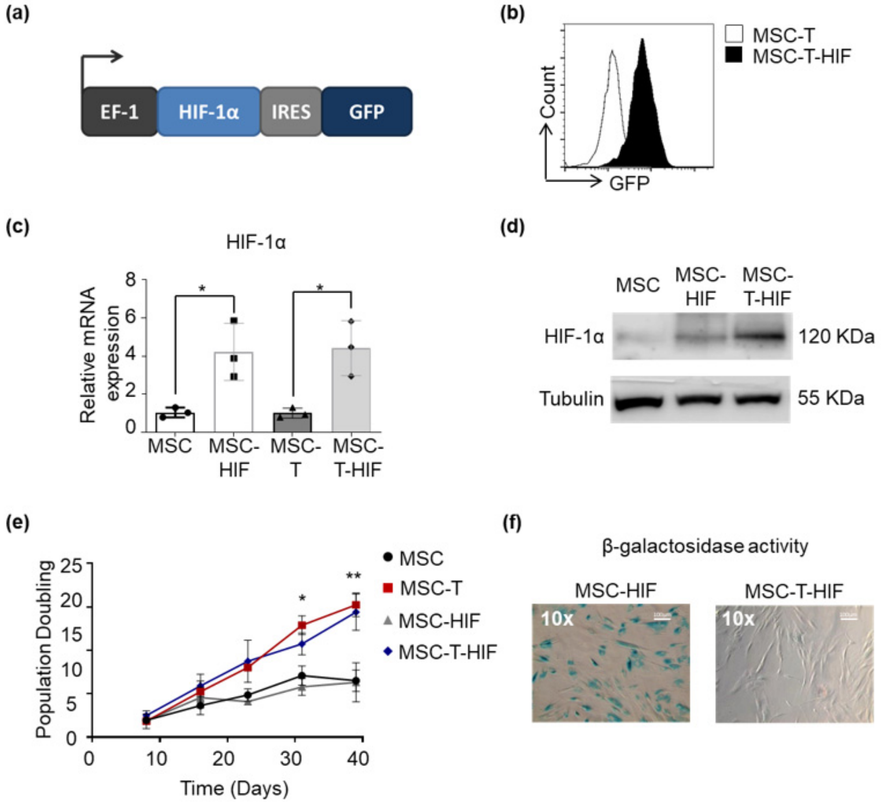

Scheme 2. Overexpression of HIF-1α in MSCs-T does not affect cell lifespan. a) Schematic representation of pWPI-HIF-1α-GFP lentiviral vector. b) Lentiviral transfection with pWIPI-HIF-GFP (MSCs-T-HIF) detected by flow cytometric analysis of GFP. c) HIF-1α gene expression levels detected by RT-qPCR in MSC-HIF (white bars) and MSC-T-HIF (stripped white bars). The expression levels of the target gene in each sample were normalized against GAPDH expression. MSCs were transduced with the empty pWPI-GFP vector. Graphs represent mean ± SD of fold change of 3 independent experiments. Paired t-test was used for statistics. d) Representative western blot of HIF-1α protein in MSC transduced with pWPI-GFP, pWPI-HIF-1α-GFP or pWPI-HIF-1α-GFP followed by hTERT lentiviral vectors; α-tubulin was used as a protein loading control. e) Proliferation assay. MSC (black), MSC-HIF (grey), MSCT-T (red) and MSC-T-HIF (blue) growth curve; represented by doubling population time. Each point represents mean ± SD of 3 independent experiments. Paired t-test was used for statistics. f) Representative image of senescence associated β-galactosidase detection in MSC-HIF (left) and MSC-T-HIF (right) at passage 14. * $p < 0.05$. .

(a)

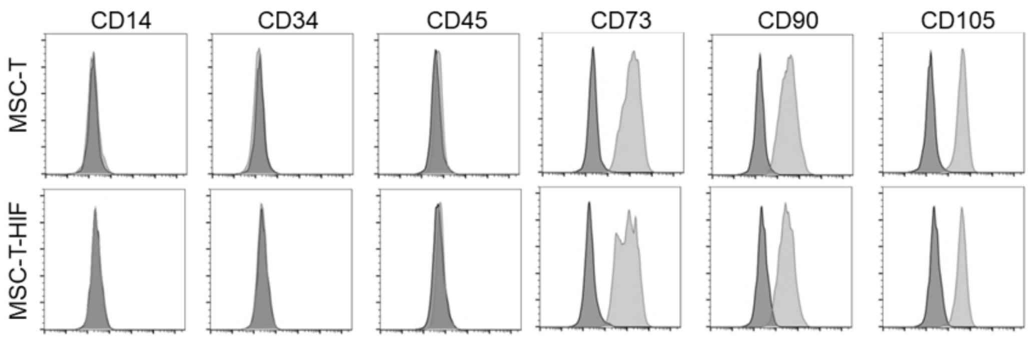

Scheme 3. Genetic modification does not affect characteristic MSC surface markers. Cell surface marker profile of different MSC lines.

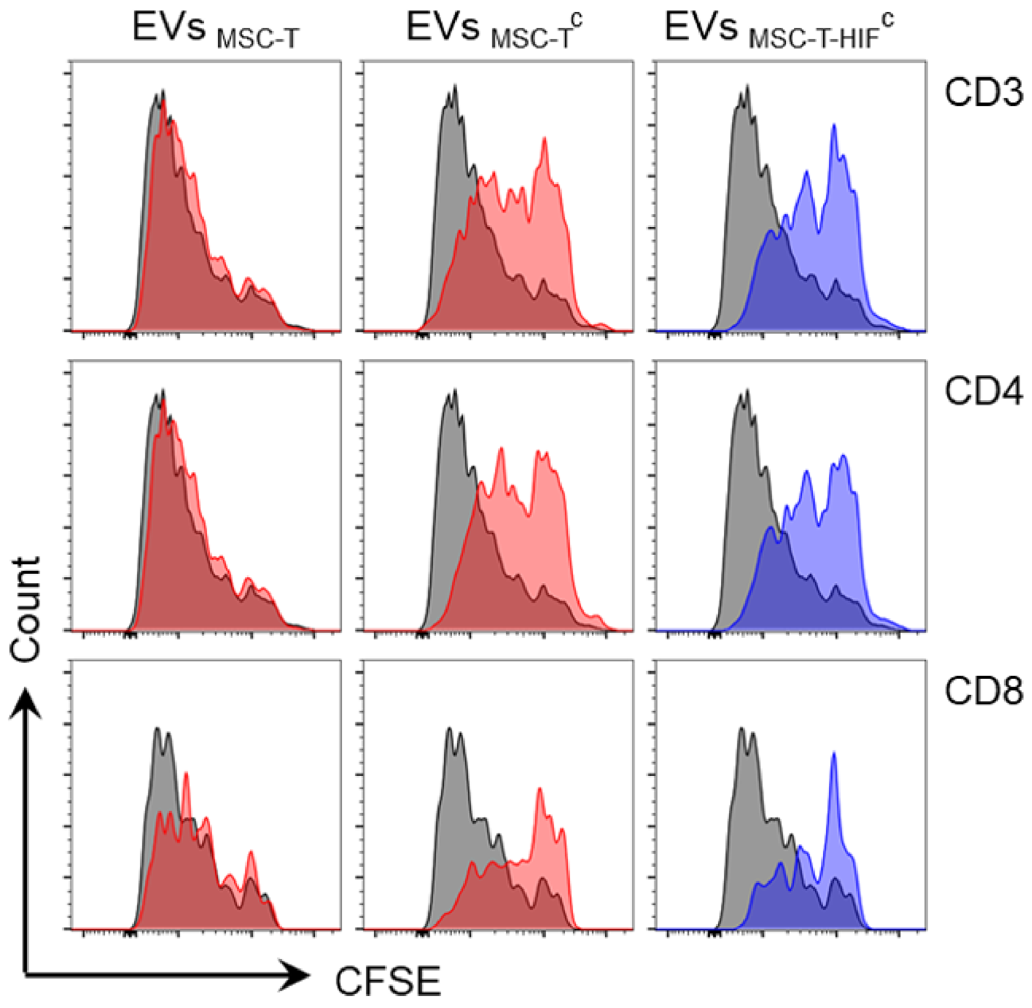

Scheme 4. EVs from MSC-T-HIFc display enhanced immunosuppression properties. Representative flow cytometry plots of CFSE dilution in CD3+ cells activated with CD3/CD28 beads and treated with different EVs (colored) or without EVs (grey).

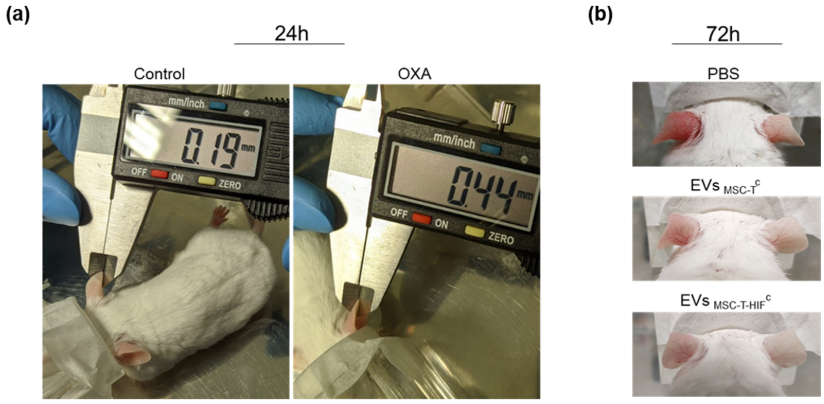

Scheme 5. DTH measurements. a) Images illustrating the procedure used to measure ear swelling using a digital caliper in a control or oxazolone treated mice. b) Representative images of ears in oxazolone treated mice, 72h after subcutaneous infusion of PBS, EVMSC-T and EVMSC-T-HIFc.
